# Antibody Conjugated PLGA Nanocarriers and Superparmagnetic Nanoparticles for Targeted Delivery of Oxaliplatin to Cells from Colorectal Carcinoma

**DOI:** 10.3390/ijms23031200

**Published:** 2022-01-21

**Authors:** Alma Lucia Villela Zumaya, Silvie Rimpelová, Markéta Štějdířová, Pavel Ulbrich, Jarmila Vilčáková, Fatima Hassouna

**Affiliations:** 1 Faculty of Chemical Engineering, University of Chemistry and Technology Prague, 166 28 Prague, Czech Republic; zumayaa@vscht.cz (A.L.V.Z.); stejdirm@vscht.cz (M.Š.); 2 Faculty of Food and Biochemical Technology, University of Chemistry and Technology Prague, 166 28 Prague, Czech Republic; silvie.rimpelova@vscht.cz (S.R.); ulbrichp@vscht.cz (P.U.); 3 Faculty of Technology, Tomas Bata University, 760 01 Zlín, Czech Republic; vilcakova@utb.cz

**Keywords:** PLGA nanoparticles, iron oxide nanoparticles, antibody, colorectal cancer, oxaliplatin, drug delivery, targeted delivery

## Abstract

Anti-CD133 monoclonal antibody (Ab)-conjugated poly(lactide-co-glycolide) (PLGA) nanocarriers, for the targeted delivery of oxaliplatin (OXA) and superparamagnetic nanoparticles (IO-OA) to colorectal cancer cells (CaCo-2), were designed, synthesized, characterized, and evaluated in this study. The co-encapsulation of OXA and IO-OA was achieved in two types of polymeric carriers, namely, PLGA and poly(lactide-co-glycolide)-poly(ethylene glycol) (PLGA-PEG) by double emulsion. PLGA_IO-OA_OXA and PEGylated PLGA_IO-OA_OXA nanoparticles displayed a comparable mean diameter of 207 ± 70 nm and 185 ± 119 nm, respectively. The concentration of the released OXA from the PEGylated PLGA_IO-OA_OXA increased very rapidly, reaching ~100% release after only 2 h, while the PLGA_IO-OA_OXA displayed a slower and sustained drug release. Therefore, for a controlled OXA release, non-PEGylated PLGA nanoparticles were more convenient. Interestingly, preservation of the superparamagnetic behavior of the IO-OA, without magnetic hysteresis all along the dissolution process, was observed. The non-PEGylated nanoparticles (PLGA_OXA, PLGA_IO-OA_OXA) were selected for the anti-CD133 Ab conjugation. The affinity of Ab-coated nanoparticles for CD133-positive cells was examined using fluorescence microscopy in CaCo-2 cells, which was followed by a viability assay.

## 1. Introduction

According to the Global Cancer Observatory, colorectal cancer is the third most commonly diagnosed cancer, with ca. 1.93 million new cases diagnosed in 2020, making it the second major cause of cancer deaths [1,2]. This disease can be associated with socioeconomic development around the world, as changes in lifestyle and nutrition are key components on the impact on the intestinal microbiota associated with the risk of developing colorectal cancer [3,4]. The first-line treatment of colorectal cancer began in 1957, with chemotherapy based on 5-fluorouracil (5-FU) as an inhibitor of thymidylate synthase, which leads to the blocking of tumor cell division [5,6]. Later, further key progress was achieved, where the inhibition of thymidylate synthase could be potentiated by co-administration with reduced folate (e.g., leucovorin [5-formyl tetrahydrofolate]) as it increased the binding efficacy of 5-FU [7,8]. In the early 2000s, the co-administration of irinotecan (prodrug) and/or oxaliplatin (OXA) with 5-FU/leucovorin was associated with further enhanced cytotoxicity against metastatic colorectal cancer cells, thus preventing the DNA from unwinding and ultimately, cell death [9,10]. The next step was the development of treatments that target the specific genetic mutations of tumors. The introduction of monoclonal antibodies enabled this possibility. Bevacizumab, a humanized monoclonal antibody, was introduced into clinical trials in 2004, to target angiogenesis by the inhibition of the vascular endothelial growth factor [11]. A different receptor, the epidermal growth factor, was also targeted for colorectal cancer treatment using monoclonal antibodies, enabling the growth inhibition of cancer cells; the antibodies cetuximab and panitumumab were the first therapeutic agents used in clinical trials for colorectal cancer [11,12,13]. Although great advances against colorectal cancer have been developed over the past decades, the need to overcome adverse reactions, suppress dose-limiting toxicities, improve the stability, pharmacokinetics, bioavailability and non-specific biodistribution are key factors that need to be addressed. An alternative to overcome these issues is by the application of nanomedicine [14], which entails the use of delivery systems engineered with specific materials at a controlled size, in the range of one to hundreds of nanometers that can carry the therapeutic agent to the desired target site. Several types of delivery systems have been investigated as colorectal cancer treatment, such as polymeric nanoparticles, lipid nanoparticles, polymeric micelles, dendrimers, as well as gold, silver, and magnetic nanoparticles [15,16,17,18,19].

The ability to encapsulate therapeutic agents for colorectal cancer, such as the platinum compound OXA, can suppress dose-limiting toxicities and bioavailability during treatment. OXA is a third-generation platinum-based chemotherapeutic agent, which is an oxalato(*trans*-l-1,2-cyclohexanediamine)platinum(II), with a better safety profile than first-generation platinum agents (e.g., cisplatin) [20]. However, even though OXA has demonstrated significant activity in clinical practice against colorectal cancer, it can induce peripheral neurotoxicity (IPN) in the form of acute and chronic clinical syndrome [21]. In fact, cases of IPN caused by cumulative doses of OXA remain a treatment-limiting factor, which has raised great interest in the design of polymeric nanoparticle-based OXA delivery systems, with controlled and targeted delivery to address this issue [22,23,24]. To build a polymeric nanoparticle-based OXA delivery system useful for cancer treatment, biodegradability and biocompatibility of the polymer are important requisites to consider. Owing to their biocompatibility, biodegradability, and low toxicity, polymers from the group of aliphatic polyesters, such as poly(lactide-co-glycolide) (PLGA), have emerged as a promising class of polymers in the development of nanoparticles for the treatment of various conditions, including cancer [25,26,27]. PLGA has been particularly relevant in the preparation of nanoparticles for drug delivery and utilized in market products, authorized by the U.S. Food and Drug Administration [25,28]. Not to mention that the PLGA degradation rate can be controlled by adjusting the molecular weight, the enantiomeric nature of the lactide monomer (namely, L- and D-lactide), and the molar ratio between the monomer units (lactide and glycolide units) during the polymerization [29]. In addition, the chemical reactivity of the surface of PLGA nanoparticles enables the adaptation of diverse functionalities, such as PEGylation [30], and conjugation with specific recognition ligands to achieve active targeted delivery [31].

Polymeric nanocarriers can also make it possibile to deliver therapeutic agents with different properties, within a multicomponent single particle, to substantially enhance the treatment efficacy [32,33,34,35]. Functionalities, such as drug delivery, targeting, magnetic hyperthermia, imaging, and radiotherapeutics may be combined in such nanocarriers. In this regard, superparamagnetic iron oxide (IO) nanoparticles have unique properties, due to their small size, magnetic anisotropy, magnetoresistance, magnetocaloric effect, the feasibility of large scale, and surface chemistry modification [36,37,38]. IO nanoparticles might be employed as hyperthermia agents, by which they can deliver toxic amounts of heat to tumors, or as radiotherapy enhancement agents. IO nanoparticles can also act as magnetic resonance contrast agents.

Finally, to further improve the efficacy of the therapy, targeting ligands can be conjugated into the multicomponent drug delivery system. Among the ligands, monoclonal antibodies (Abs) have been the most widely studied, since they can actively target a variety of tumor types, as the antibody can recognize biological macromolecules (if properly selected) in healthy and diseased cells [39,40]. For example, the introduction of an anti-CD133 Ab has been a pivot for targeted treatment against cancer, as the cell surface marker CD133 (prominin-1) has been identified in various types of cancers, such as brain, prostate, and colorectal cancer. The cell surface marker, CD133, is a five-domain transmembrane glycoprotein that is present in cancer stem cells (CSC). The CSC are a group of cancer cells associated with the progression of tumor growth and metastasis and have been identified in colorectal cancer [41].

Therefore, the aim of this study focused on the development of Ab-targeted multicomponent and multifunctional drug delivery nanoparticle conjugates, using superparamagnetic IO nanoparticles and OXA, both encapsulated in PLGA and PEGylated PLGA nanoparticles by the double emulsion method [42]. Surface modification of PLGA nanoparticles was achieved by conjugation with an anti-CD133 monoclonal Ab (conjugated to Alexa Fluor 488), which can enable it to target the PLGA nanoparticles with its cargo (e.g., OXA) and, thus, enhance the cargo efficacy, improving distribution and concentration at the site of action in the colorectal cancer cells [39,43]. Using this approach, it is expected on one hand, to suppress the IPN caused by cumulative doses of OXA, which remains a treatment-limiting factor, and on the other hand, to enhance the treatment efficacy via delivery of two therapeutic agents (i.e., OXA and IO nanoparticles) with different properties, within a multicomponent single particle. The physicochemical properties, morphology, zeta potential, and size distribution of the nanoparticles were studied. Furthermore, the drug loading, encapsulation efficiency, and drug release kinetics were carried out in a simulated physiological environment. In addition, the affinity of the Ab-coated nanoparticles was examined using live-cell fluorescence microscopy, using CaCo-2 and MRC-5 cells, as well as by metabolic activity and viability assay.

## 2. Results

### 2.1. Physicochemical Characteristics of the Iron Oxide Nanoparticles

**Figure 1:**
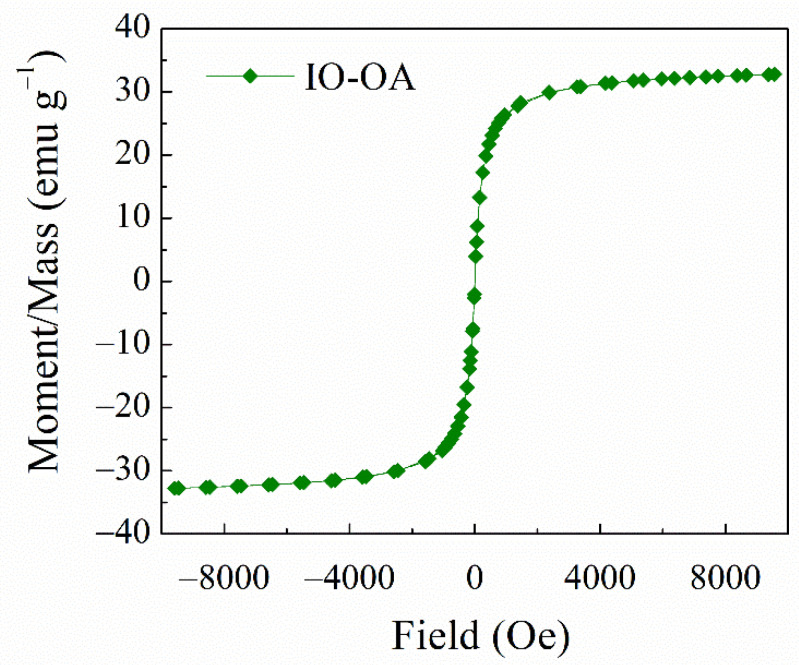
Magnetic properties at room temperature (25 °C) of oleic acid-coated iron oxide nanoparticles (IO-OA) synthesized by co-precipitation. The measurement was performed in the magnetic field from −10 kOe to +10 kOe.

The preparation of oleic acid-coated iron oxide nanoparticles (IO-OA), with a narrow size distribution, was achieved by the co-precipitation method using oleic acid (OA) as the coating agent and further dispersed in an organic phase (e.g., chloroform). According to the DLS measurements, IO-OA displayed a mean diameter of 20 ± 4 nm and zeta potential of −36 ± 4 mV (Table 1). The negative zeta potential value indicates the coating of IO with OA, which confers them good colloidal stability and it is in agreement with our previous study [33]. XRD analysis confirmed the superparamagnetic structure of IO-OA nanoparticles, i.e., Fe_3_O_4_ (Figure S1), where X-ray peaks were identified similarly to previous work [33]. The superparamagnetic properties of the prepared nanoparticles were analyzed by VSM with a coercivity value of 3.91 Oe and a saturation magnetization (Ms) of 0.43 emu/g. The nanoparticles showed paramagnetism behavior without magnetic hysteresis at room temperature (25 °C), as depicted in Figure 1, which makes them act as good MRI contrast agents or suitable for treatment with magnetically mediated hyperthermia [38,44].

### 2.2. Chemical Structure of the Diblock Copolymer PLGA-PEG-NH_2_

**Figure 2:**
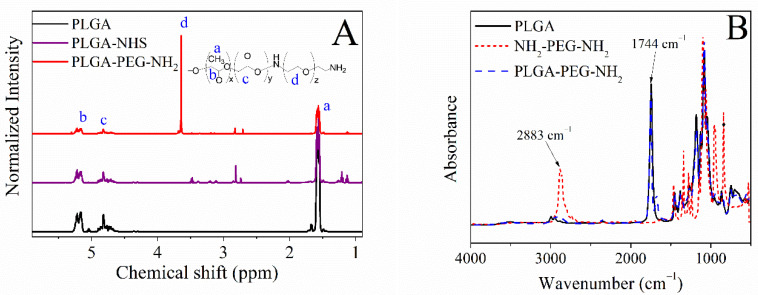
(**A**) ^1^H-NMR spectra of the PLGA, activated PLGA and diblock copolymer PLGA-PEG-NH_2_, and (**B**) FTIR spectra of PLGA, PEG diamine, and synthesized diblock copolymer PLGA-PEG-NH_2_.

The poly(lactide-co-glycolide)-poly(ethylene glycol) bearing amino end group (PLGA-PEG-NH_2_) diblock copolymer was synthesized as a step towards PEGylation of the PLGA nanoparticles to enhance their particle stability, prolong their circulation time when administered in the body and reduce their intermolecular aggregation through inter-particle steric repulsion [45,46,47]. Following the procedure reported in our previous work [33], the activation of the PLGA matrix was achieved by carbodiimide chemistry, using DCC/NHS and the subsequent attachment of the PEG diamine (NH_2_-PEG-NH_2_). The conjugation of the PEG diamine to the PLGA backbone was revealed by ^1^H-NMR, FTIR, and SEC. ^1^H-NMR spectra, depicted in Figure 2A, attest to the covalent attachment of the PEG diamine onto PLGA end chains. The chemical shifts identified at 1.59 ppm, 3.61–3.64 ppm, 4.50–4.90 ppm, and 5.13–5.30 ppm correspond to the -CH_3_ of the lactide, -CH_2_- of the ethylene glycol, -CH_2_- of the glycolide, and -CH- of the lactide, respectively. FTIR analysis further confirmed the formation of the diblock copolymer (Figure 2B), where the absorption band at 2883 cm^−1^ ascribed to the C-H stretching vibration of the methylene group of PEG chains was noticed in the PLGA-PEG-NH_2_ spectrum, confirming the reproducibility of the synthesis and in agreement with our reported work [33].

### 2.3. Multicomponent Drug Delivery Systems: Mean Size, Morphology, and Surface Properties

The multicomponent drug delivery systems, namely, PLGA_IO-OA_OXA, and PEGylated PLGA_IO-OA_OXA, were produced by the double emulsion method [42]. As a frame of reference, polymeric nanoparticles loaded with a single therapeutic agent were also prepared, namely, PLGA_IO-OA, PLGA_OXA, PEGylated PLGA_IO-OA, and PEGylated PLGA_OXA.

**Figure 3:**
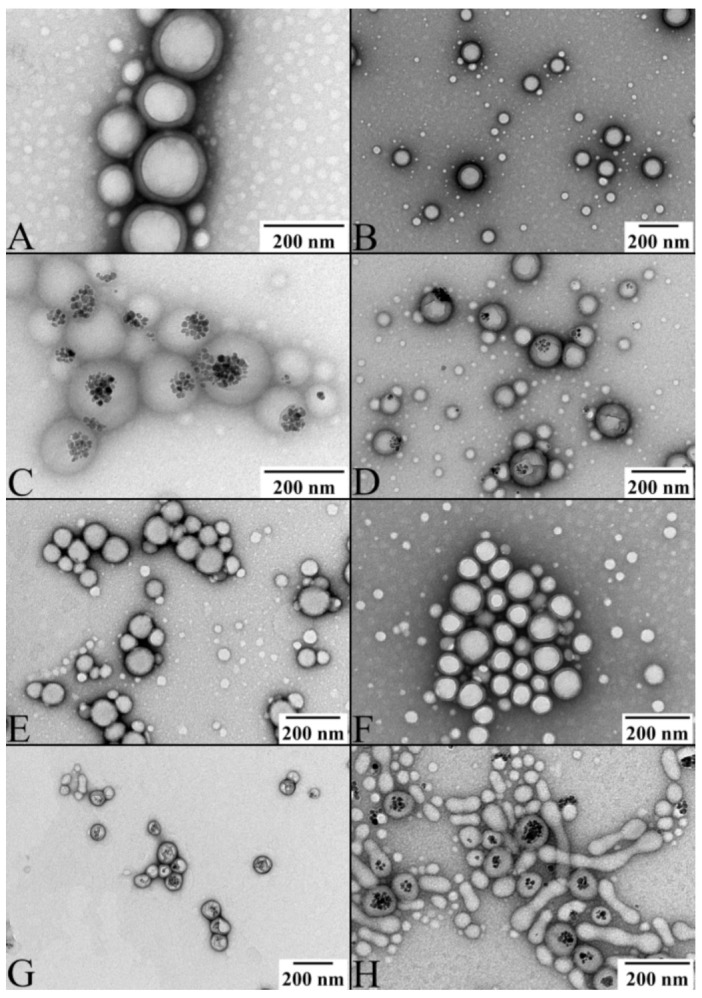
TEM pictures of (**A**) PLGA nanoparticles; (**B**) oxaliplatin-loaded PLGA nanoparticles (PLGA_OXA); (**C**) oleic acid-coated iron oxide-loaded PLGA nanoparticles (PLGA_IO-OA); (**D**) PLGA-based multicomponent delivery systems (PLGA_IO-OA_OXA); (**E**) PEGylated PLGA nanoparticles; (**F**) oxaliplatin-loaded PEGylated PLGA nanoparticles (PEGylated PLGA_OXA); (**G**) oleic acid-coated iron oxide-loaded PEGylated PLGA nanoparticles (PEGylated PLGA_IO-OA) and (**H**) PEGylated PLGA-based multicomponent delivery systems (PEGylated PLGA_IO-OA_OXA).

The size distribution of all the nanoparticles measured by DLS is depicted in Figure S2 and the average values are summarized in Table 1. Based on the DLS measurements, all produced nanoparticles, i.e., PLGA, PLGA_OXA, PLGA_IO-OA, PLGA_IO-OA_OXA, PEGylated PLGA, PEGylated PLGA_OXA, PEGylated PLGA_IO-OA, and PEGylated PLGA_IO-OA_OXA exhibited a comparable mean diameter within the standard deviation. For instance, PLGA_IO-OA_OXA and PEGylated PLGA_IO-OA_OXA displayed a mean diameter of 207 ± 70 nm and 185 ± 119 nm, respectively. These results show that the encapsulation of OXA and/or IO-OA and the PEGylation process do not affect, statistically, the hydrodynamic size of the nanoparticles (*p* ≥ 0.05) [48]. The mean size of nanoparticles is usually affected rather by the sonication power and time, and by the amount of the surfactant [30]. Conversely, the zeta potential values varied between the PEGylated and non-PEGylated analogs, e.g., −8 ± 7 mV for PLGA_IO-OA_OXA and 4 ± 3 mV for PEGylated PLGA_IO-OA_OXA. This discrepancy in the zeta potential values is associated with the nature of the functional groups present on the surface of the nanoparticles. The inversion of the zeta potential values from negative in PLGA-based nanoparticles to positive in the PEGylated analogs is consistent with the presence of amine groups in the latter from the PLGA-PEG diblock copolymer. It should be noted that the zeta potential values of the nanoparticles remained the same within the standard deviation in the PBS buffer. Examples of TEM images of the prepared nanoparticles are depicted in Figure 3. All of them exhibited a spherical shape in size ranges of hundreds of nanometers. Image treatment of TEM pictures using ImageJ software indicated a smaller average diameter (Table S1) of the nanoparticles compared to the DLS measurements. This discrepancy is associated with the principle of analysis of each method. Indeed, DLS measures the mean hydrodynamic diameter of nanoparticles dispersed in an aqueous solution, while TEM analysis allows us to determine the average diameter of dehydrated nanoparticles. They also showed the encapsulation of the IO-OA within the polymeric nanoparticles (Figure 3C,D,G,H). It is worth noting that the high PDI presented in PEG-conjugated nanoparticles is associated with their slight lack of stability, compared to their non PEG-conjugated analogs. Indeed, the presence of PEG on the surface of PLGA may lead to a coalescence-like behavior between some individual nanoparticles, due to the low zeta-potential (i.e., between 0 and 5 mV). This behavior can be also seen in the TEM images (Figure 3G,H).

**Figure 4:**
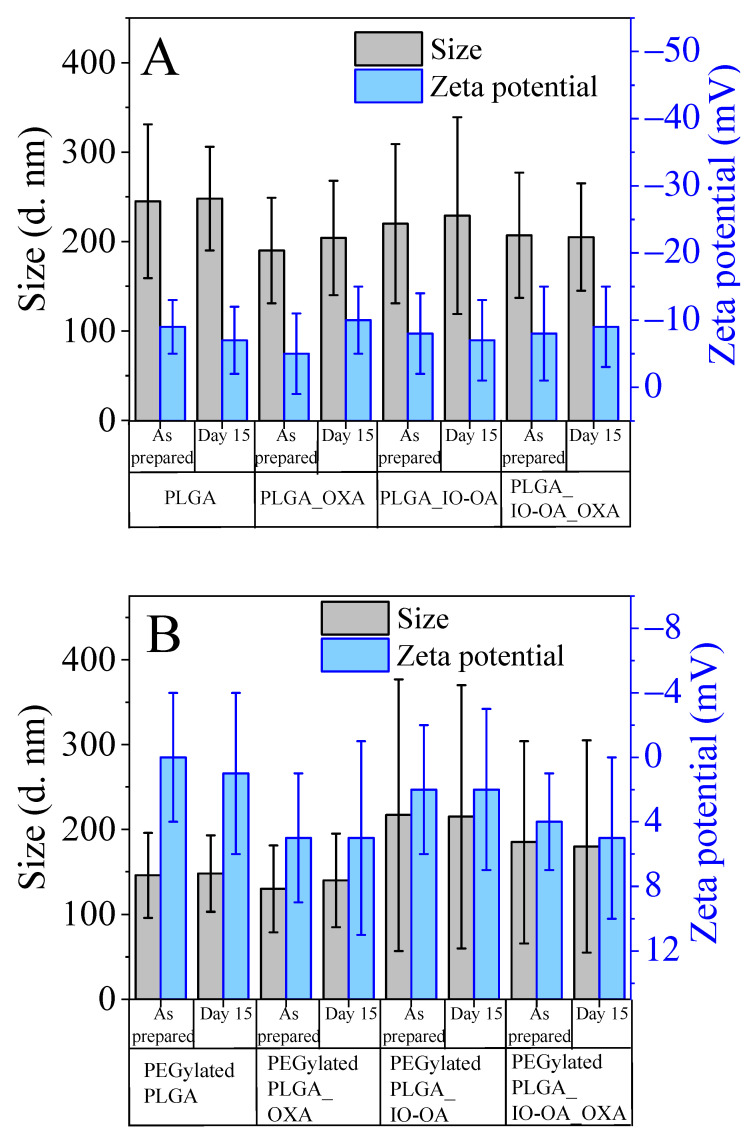
Size and zeta potential measurement at day one and day 15 of (**A**) PLGA nanoparticles (PLGA), oxaliplatin-loaded PLGA nanoparticles (PLGA_OXA), oleic acid-coated iron oxide-loaded PLGA nanoparticles (PLGA_IO-OA), PLGA-based multicomponent delivery systems (PLGA_IO-OA_OXA) and their PEGylated analogs (**B**). The ratio of IO-OA:PLGA, OXA:PLGA, and their PEGylated analogs is equal to 1:10.

The colloidal stability of the nanoparticles was monitored over 15 days, as can be seen in Figure 4. The mean diameter, as well as the zeta potential values for all nanoparticles, including the multicomponent drug delivery systems, was maintained without any significant changes (*p* ≥ 0.05).

### 2.4. Drug Loading and Drug Release Kinetics

**Figure 5:**
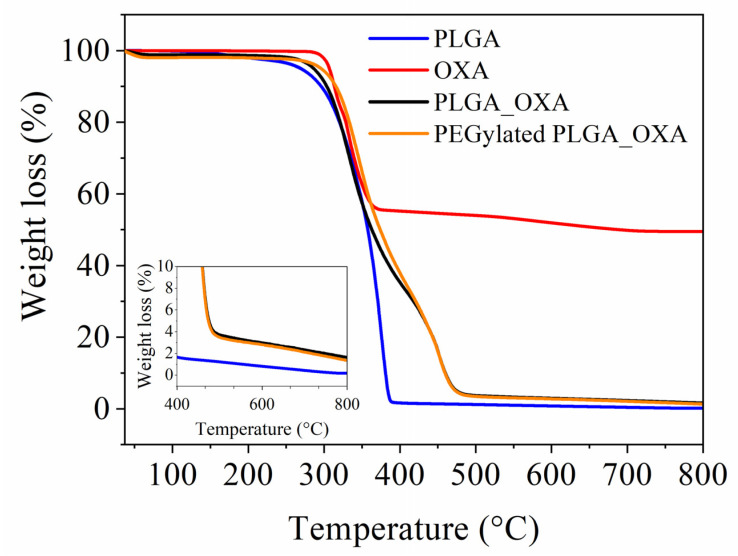
TG curves of pristine PLGA matrix, oxaliplatin (OXA), oxaliplatin-loaded PLGA nanoparticles (PLGA_OXA) and oxaliplatin-loaded PEGylated PLGA nanoparticles (PEGylated PLGA_OXA) under nitrogen atmosphere from room temperature (25 °C) to 800 °C at a heating rate of 10 °C·min^−1^.

The determination of the content of OXA in the drug delivery nanoparticles is crucial for defining the drug loading and encapsulation efficiency. The UV-visible spectrophotometer failed in determining the drug loading with high accuracy, since OXA absorbs in the same UV region as the PLGA polymer, in the range of 180–217 nm (Figure S3). The use of multiple extraction steps to isolate OXA from the polymer with high extraction efficiency was unsuccessful. To overcome this issue, TGA analysis was used to quantify the OXA encapsulated in the nanoparticles (Figure 5), since this drug possesses Pt in its chemical structure, which remains undegradable during the thermal cycle up to 800 °C (the highest applied temperature). According to the TG curve of pure OXA, the total weight loss corresponding to the decomposition of the organic part reaches 50% at 800 °C and, hence, the remaining 50% of the mass corresponds to Pt [49]. As the total weight loss of the PLGA and PLGA-PEG-NH_2_ matrices is 100% at 800 °C, hence the TG curves of PLGA_OXA and PEGylated PLGA_OXA nanoparticles, the remaining mass of 2% at 800 °C corresponds to the Pt present in the nanoparticles, which represents half of the total weight fraction of OXA in the nanoparticles. The total mass of OXA encapsulated in the nanoparticles corresponds to 4%. Based on the TGA results, a drug loading of 22% and an encapsulation efficiency of 44% were calculated in both PLGA_OXA and PEGylated PLGA_OXA. The PEGylation of the PLGA nanoparticles does not have an impact on the encapsulation efficiency, which is in good agreement with our previous study using a different drug [33]. TGA analysis cannot be used to determine the drug loading of OXA in the multicomponent PLGA_IO-OA_OXA and PEGylated PLGA_IO-OA_OXA, since both contain IO, as Pt does not decompose at the maximum applied temperature (800 °C). Nevertheless, our previous study clearly showed, with very good reproducibility of the experiments, that the encapsulation of an additional therapeutic agent (i.e., IO) besides the drug, within a PLGA/PEGylated PLGA nanocarrier, does not affect the encapsulation efficiency [33]. Based on these findings, it is reasonable to conclude that the drug loading and encapsulation efficiencies in PLGA_IO-OA_OXA and PEGylated PLGA_IO-OA_OXA nanoparticles were the same as for PLGA_OXA and PEGylated PLGA_OXA nanoparticles.

**Figure 6:**
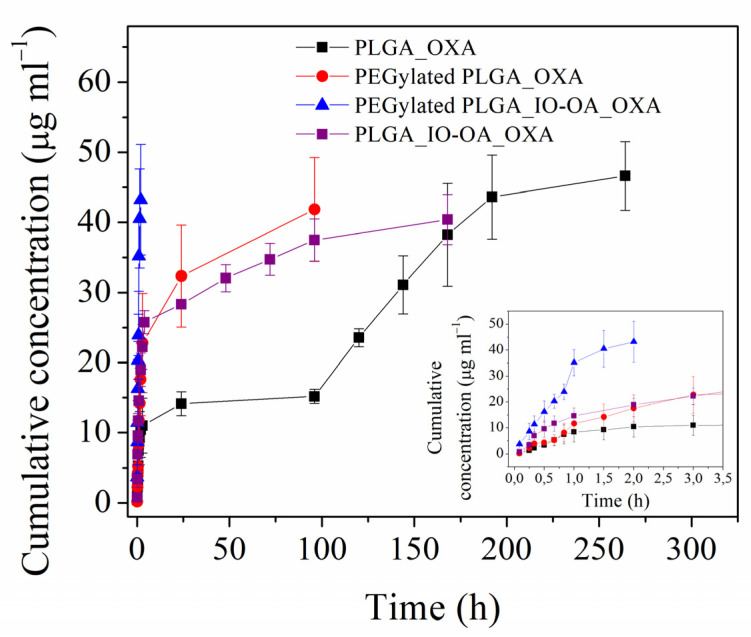
Drug release profiles of oxaliplatin-loaded PLGA nanoparticles (PLGA_OXA), oxaliplatin-loaded PEGylated PLGA nanoparticles (PEGylated PLGA_OXA), PLGA-based multicomponent delivery systems (PLGA_IO-OA_OXA) and PEGylated PLGA-based multicomponent delivery systems (PEGylated PLGA_IO-OA_OXA) in PBS pH 7.4 at 37 °C.

Prior to Ab conjugation, the drug release profiles of the multicomponent drug delivery systems PLGA_IO-OA_OXA and PEGylated PLGA_IO-OA_OXA were examined and compared to those of their drug-loaded nanoparticle analogs free of IO-OA, namely, PLGA_OXA and PEGylated PLGA_OXA. The dissolution tests were monitored over several days in PBS at 37 °C (Figure 6 and Table S2). The concentration of the released OXA from the PEGylated PLGA_IO-OA_OXA increased very rapidly during the first minutes, reaching ~100% release (40 ± 4 µg·mL^−1^) after only 2 h. In PEGylated PLGA_ OXA free of IO-OA, the drug release profile was characterized by an initial burst release of ~75%, which occurred during the first 2 h, followed by a slower and controlled drug release, until reaching ~100% release (43 ± 8 µg·mL^−1^) after 96 h. The super-fast and non-controlled OXA release in PEGylated PLGA_IO-OA_OXA, taking place during the burst release phase, is probably associated with the hydrophilic nature of OXA, the PEGylation of the nanoparticles, and the presence of IO-OA [33,34]. Owing to their hydrophilic nature, OXA may preferentially locate close to or within the PEG shell. This phenomenon might be enhanced in the presence of another hydrophobic therapeutic agent, such as IO-OA, in the nanoparticles, as they can induce more favorable interactions than OXA with the PLGA matrix. Conversely, PLGA_OXA and PLGA_IO-OA_OXA displayed a slower and more sustained drug release compared to their PEGylated analogs. The dissolution profile of PLGA_IO-OA_OXA exhibited a prominent initial burst release of about 45% during the first 2 h, which was followed by a slower release, until reaching ~100% release (40 ± 4 µg·mL^−1^) after 168 h. Interestingly, PLGA_OXA free of IO-OA displayed a triphasic profile, characterized by an initial burst release of about 20% during the first 2 h. This is followed by diffusion, swelling, and lastly an accelerated drug release phase, caused by the polymer degradation, attaining ~100% release (45 ± 4 µg·mL^−1^) after 264 h [50]. The faster drug release observed in the presence of IO-OA (i.e., PLGA_IO-OA_OXA) is caused by the catalytic activity of the magnetic nanoparticles, leading to faster degradation of the PLGA matrix. Moreover, the faster drug release, noticed in the PEGylated nanoparticles compared to their non-PEGylated analogs, is due to the hydrophilic nature of PEG chains that thereby facilitate the water uptake and accelerate the degradation of the PLGA matrix by hydrolysis. The effect of PEGylation on the degradation kinetics of PLGA during the drug release process has already been described in the literature [33,51]. Therefore, for a controlled OXA release, non-PEGylated PLGA multicomponent systems are more suitable. Based on these findings, Ab conjugation was carried out on PLGA_IO-OA_OXA.

**Figure 7:**
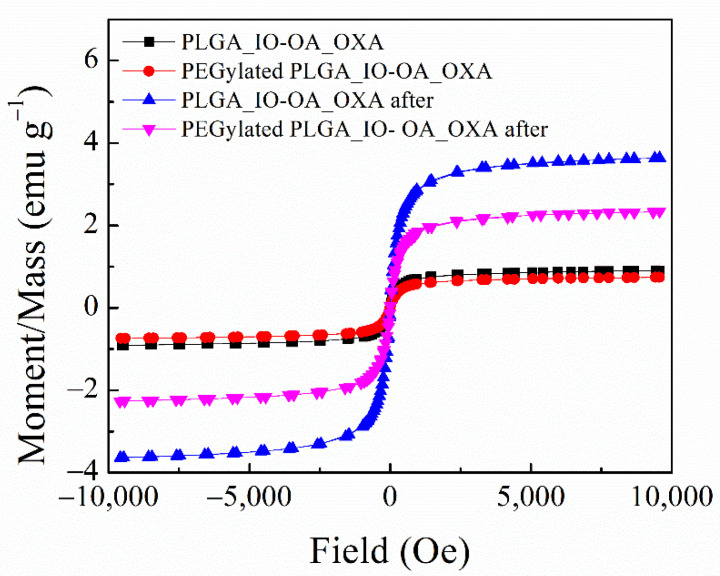
Magnetization curves for the multicomponent PLGA based delivery system (PLGA_IO-OA_OXA) and its PEGylated analog (PEGylated PLGA_IO-OA_OXA) before and after dissolution tests measured at room temperature (25 °C) in the magnetic field from −10 kOe to +10 kOe.

Besides the drug release performance of the multicomponent drug delivery systems, i.e., PLGA_IO-OA_OXA and PEGylated PLGA_IO-OA_OXA, their magnetic properties were examined before and after dissolution tests (Figure 7). The magnetization curves of both PLGA_IO-OA_OXA and PEGylated PLGA_IO-OA_OXA present zero remanence and zero coercivity before, as well as after, the dissolution tests. The saturation magnetization for PLGA_IO-OA_OXA reached a value of 0.0019 emu·g^−1^ with a coercivity of 2.89 Oe, and at the end dissolution test, this value increased to 0.0091 emu·g^−1^ with a coercivity of 3.81 Oe. Likewise, the same trend was observed in the case of PEGylated PLGA_IO-OA_OXA, for which an increase in the saturation magnetization value was detected, from 0.0021 emu·g^−1^ (coercivity of 3.07 Oe) before dissolution tests to 0.0051 emu·g^−1^ (coercivity of 3.61 Oe) after 96 h of drug release (the end of dissolution test). The increase in both saturation magnetization and coercivity values after complete drug release can be explained by the degradation of the polymeric matrix, leading to the apparent increase in the concentration of IO-OA in the remaining solid content. The preservation of the superparamagnetic behavior of the IO-OA, without magnetic hysteresis all along the dissolution process, makes the multicomponent drug delivery systems suitable for treatment with magnetically mediated hyperthermia, even after the drug release is completed.

### 2.5. In Vitro Antitumor Activity of PLGA_IO-OA_OXA_Ab

**Figure 8:**
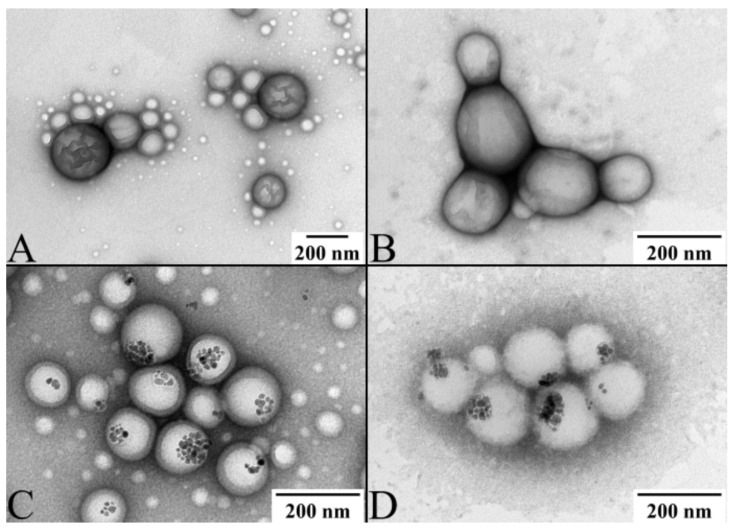
Transmission electron microscopy images of the oxaliplatin-loaded PLGA nanoparticles, PLGA_OXA (**A**); anti-CD133 antibody conjugated oxaliplatin loaded PLGA nanoparticles, PLGA_OXA_Ab (**B**); multicomponent PLGA based delivery system, PLGA_IO-OA_OXA (**C**) and anti-CD133 antibody conjugated multicomponent PLGA-based delivery system, PLGA_IO-OA_OXA_Ab (**D**).

To develop a multicomponent and multifunctional delivery system, capable of both drug delivery and cellular targeting, Ab-conjugated PLGA nanoparticles were prepared by covalent attachment of the anti-CD133 Ab to the surface of PLGA_IO-OA_OXA, further labeled as PLGA_IO-OXA_Ab. For comparison, PLGA_OXA_Ab nanoparticles were also prepared. As can be seen in Table 1, after Ab conjugation, the mean diameter, as well as the zeta potential of PLGA_OXA_Ab and PLGA_IO-OA_OXA_Ab, were comparable to their unconjugated analogs when taking into account the standard deviation. The coating of the nanoparticle surface by Ab is apparent in the TEM pictures in Figure 8B,C.

**Figure 9:**
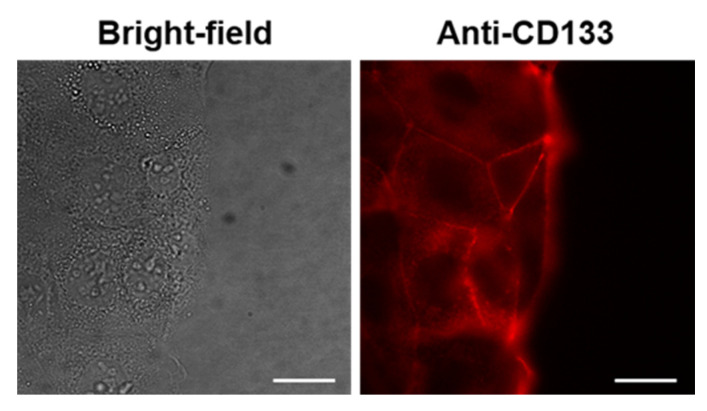
Fluorescence microscopy images of human cells derived from colorectal carcinoma (CaCo-2) stained with anti-CD133 antibody conjugated to Atto 565 for 30 min. Left—a bright-field image of the cells. Right—fluorescence emission of cells stained with anti-CD133-Atto 565. The scale bars correspond to 20 µm.

Regarding in vitro experiments in human cells, first, we aimed to evaluate the binding specificity of PLGA_OXA_Ab and PLGA_IO-OA_OXA_Ab in CaCo-2 cells, which should, according to the literature, express the CD133 marker, the RNA levels of which have been documented to be pronounced in CaCo-2 cells in comparison to other cell lines [52]. Therefore, to verify that the CaCo-2 cells held their characteristics, remained unchanged, and produced the CD133 expression also on the protein level, fluorescently labeled Ab anti-CD133 binding assay was performed in these cells. The staining of this surface protein was positive, which is documented in Figure 9, with fluorescence emission detected on the cell surface, meaning that CD133 is expressed at the cellular membrane of CaCo-2 cells.

**Figure 10:**
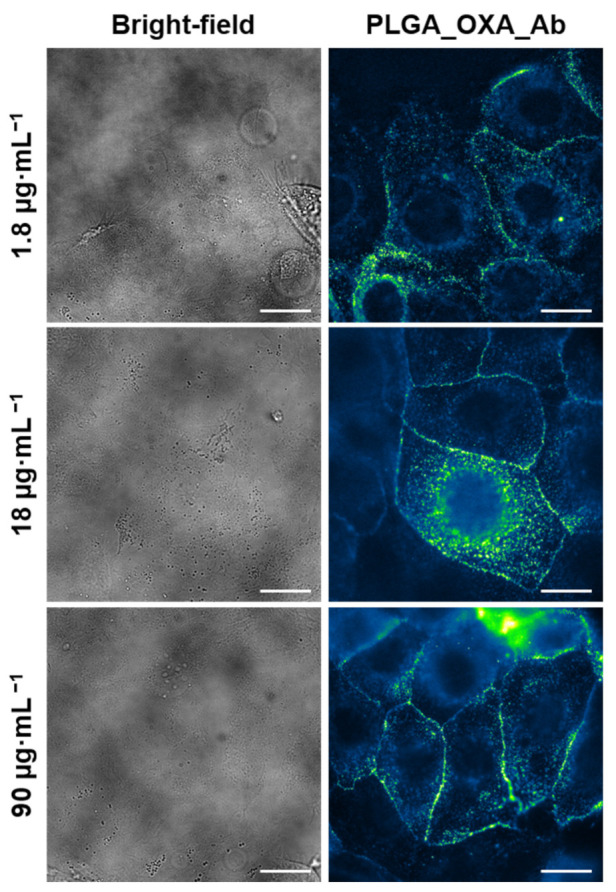
Fluorescence microscopy images of human cells derived from colorectal carcinoma (CaCo-2) treated with oxaliplatin-containing PLGA nanoparticles coated with anti-CD133 antibody conjugated to Alexa Fluor 488 (PLGA_OXA_Ab). The CaCo-2 cells were treated with 1.8–90 µg·mL^−1^ concentration of the PLGA_OXA_Ab for 30 min. Left—bright-field images of the cells. Right—fluorescence emission of cells treated with PLGA_OXA_Ab (false-colored based on the fluorescence emission intensity). The scale bars correspond to 20 µm.

Next, we aimed to investigate the localization and intracellular internalization of PLGA_OXA nanoparticles coated with anti-CD133 Ab (PLGA_OXA_Ab), which was done by binding study in living CaCo-2 cells, again using fluorescence microscopy, since this method provides a direct and facile approach for monitoring the intracellular trafficking and fate of the nanoparticles. The PLGA_OXA_Ab cell uptake was visualized by Alexa Fluor 488 (green emission), i.e., the presence of nanoparticles in CaCo-2 cells was detected using the FITC channel. Since, in general, nanoparticle uptake by cells is dose-dependent, a range of nanoparticle concentrations for the localization study was used (specifically, 1.8–90 µg·mL^−1^). As is apparent from Figure 10 (images are false-colored based on the fluorescence emission intensity), even a concentration of the nanoparticles as low as 1.8 µg·mL^−1^ was sufficient for visualization of the CD133 protein binding to the cell surface. The images were taken 30 min after the nanoparticle treatment of the CaCo-2 cells and, as can be seen, some proportion of the NPs were already internalized in the cells; therefore, except for the cell membrane, the highest fluorescence emission intensity was apparent around the cell nucleus. This effect was likely caused by the specific antigen–antibody interaction/binding, with subsequent nanoparticle internalization likely by receptor-mediated endocytosis. Therefore, here, we verified that the anti-CD133 Ab can be used for targeting CD133-positive cells.

The same data as for PLGA_OXA_Ab were gained for PLGA_IO-OA_OXA_Ab (see Figure S4 in the Supplementary Materials). This again, confirms the efficient cell surface binding of the nanoparticles and partial internalization, after only 30 min incubation. No differences were observed for nanoparticles containing iron oxide.

However, in contrast to the cancerous CaCo-2 cells, in noncancerous human primary fibroblasts (MRC-5), we have proven no PLGA_OXA_Ab binding (Figure S5 in the Supplementary Materials), as expected since MRC-5 cells should not express the CD133 protein.

**Figure 11:**
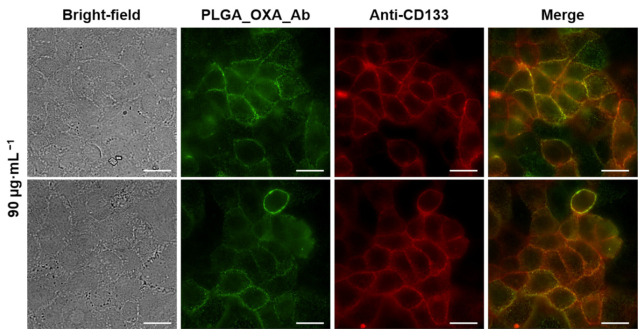
Fluorescence microscopy images of human cells derived from colorectal carcinoma (CaCo-2) treated with oxaliplatin-containing PLGA nanoparticles coated with anti-CD133 antibody conjugated to Alexa Fluor 488 (PLGA_OXA_Ab) and co-stained with anti-CD133 Atto 565. The CaCo-2 cells were treated with 90 µg·mL^−1^ concentration of the PLGA_OXA_Ab for 30 min. From left: bright-field images of the cells; fluorescence emission of cells treated with PLGA_OXA_Ab; cells stained with anti-CD133-Atto 565; merge of the fluorescence images. The scale bars correspond to 20 µm.

Next, to verify that upon PLGA_OXA_Ab and PLGA_IO-OA_OXA_Ab treatment, the detected fluorescence emission on the CaCo-2 cell membrane was caused by specific Ab binding to the CD133 antigen, a colocalization study using fluorescence microscopy was performed. In this experiment, CaCo-2 cells were treated with PLGA_OXA_Ab (90 µg·mL^−1^) for 30 min (green fluorescence emission) and co-stained with anti-CD133 Ab, conjugated to Atto 565 (red fluorescence emission). Based on the images in Figure 11, the co-localization proved to be successful. See the Supplementary Materials (Figure S6) for a zoomed section of the PLGA_OXA_Ab and PLGA_IO-OA_OXA_Ab co-localization.

**Figure 12:**
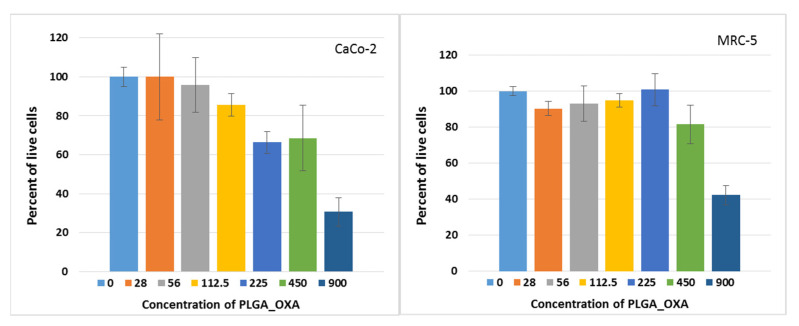
Evaluation of the percentage of living cells in vitro after PLGA_OXA treatment. Human cells derived from colorectal carcinoma (CaCo-2) and human primary fibroblasts (MRC-5) were treated with PLGA_OXA for 72 h.

As a next step, we evaluated the effect of the OXA-containing nanoparticles on the cell metabolic activity of cancerous CaCo-2 and noncancerous MRC-5 cells, using WST-1 assay and 72 h incubation. Cell treatment with the PLGA_OXA showed a dose–response relationship. The results, summarized in Table 2, show that the IC_50_ corresponded to 255 ± 11 µg·mL^−1^, in CaCo-2 cells expressing the CD133 receptor and to 525 ± 12 µg·mL^−1^, in CD133-negative noncancerous MRC-5 cells (Table 3, expressed for the OXA content only). Pure nanoparticles (PLGA_blank), not containing OXA, did not exhibit cytotoxicity and did not reach IC_50_, up to the highest evaluated concentration of 900 µg·mL^−1^. Finally, the number of living CaCo-2 cells was evaluated using calcein, after PLGA_OXA treatment (Figure 12) for 72 h. There was a slight decrease in the proportion of living cells already from the 225 µg·mL^−1^ concentration. A drop to ca. one-third of living cells, in comparison to the untreated control, was detected at the concentration of 900 µg·mL^−1^. As for the blank NPs (nanoparticles not containing OXA), no marked drop (in the range of SD) in the proportion of living cells was detected (Figure S6 in the Supplementary Materials). Therefore, these findings suggest that PLGA nanoparticles themselves are nontoxic and suitable as nanocarriers for the delivery of various drugs, as well as being suitable for Ab conjugation and, thus, specific cell targeting.

## 3. Discussion

Oxaliplatin (OXA) is considered one of the essential cytotoxic drugs for modern chemotherapy in colorectal cancer, as it has led to improvements in patients’ survival and management of the disease [53]. Other platinum derivatives, such as cisplatin and carboplatin, possess intrinsic resistance that limits their application as therapeutic agents for colorectal cancer [54,55]. The chemical structure of OXA differs from cisplatin or carboplatin, as it possesses an oxalate and a diaminocyclohexane (DACH) group (Supplementary Materials, Figure S7). Part of the cytotoxic activity of OXA has been attributed to the DACH ligand, which is retained by the activated oxaliplatin, resulting in the inhibition of the DNA synthesis by the formation of interstrand crosslink and intrastrand platinum-DNA adducts, and it appears to be more effective and cytotoxic than adducts formed from cisplatin or carboplatin [56,57]. Even though OXA has demonstrated significant activity in clinical practice against colorectal cancer, it can induce peripheral neurotoxicity (IPN) in the form of acute and chronic clinical syndrome; this is still considered the major dose-limiting factor [21]. For patients that receive a cumulative dose of OXA, it is expected that high-grade chronic IPN will occur in about 10% of patients, from receiving doses between 510–766 mg·m^−2^, while at doses higher than 1000 mg·m^−2^, the likelihood of developing IPN can reach almost 50% of treated patients [58]. Therefore, to reduce OXA IPN, its encapsulation into polymeric nanoparticles comes as a promising approach for cancer treatment. For example, in a study by Tummala et al., the encapsulation of OXA was achieved in a chitosan-based pH-responsive hybrid as a therapeutic agent [59]. In another study, Handali et al. encapsulated 5-FU and OXA into biodegradable PHBV/PLGA nanoparticles, which exhibited a higher cytotoxicity effect than free drugs on cancer cells [60,61]. Encapsulation of therapeutic drugs using biodegradable polymers, such as PLGA, has attracted attention due to its biocompatibility, non-toxicity, and non-immunogenicity [62].

In this study, the design of the drug delivery system was not only aimed at encapsulating OXA in PLGA based nanoparticles, but at combining multiple functionalities into a single system. A combination of OXA with magnetic hyperthermia, through superparamagnetic iron oxide nanoparticles (IO), could improve diagnosis and cancer therapy, as magnetic hyperthermia enables one to target the heating source at the tumor tissues, through the application of an external magnetic field [63]. Promising results have been reported using multifunctional platforms for colorectal cancer therapy [64,65]. In a study by Lima et al., the platform was designed based on polyethylene glycol–polylactic acid (PEG–PLA) nanospheres to co-deliver methotrexate and IO nanoparticles, proving increased cytotoxicity towards Caco-2 and SW-480 colon cancer cells [66]. As tumor biology is also better understood, so is the possibility to apply a targeted multifunctional drug delivery system, as this will be essential for efficient use of the resources and better outcomes for patients.

For this study, the incorporation of OXA and IO nanoparticles into PLGA-based nanoparticles with a surface targeting agent (anti-CD133 Ab) for the treatment of colorectal cancer was designed. The assembly of the multicomponent drug delivery systems was achieved by the encapsulation of oleic acid-coated iron oxide nanoparticles (IO-OA) and/or OXA into a PLGA or PLGA-PEG-NH_2_ polymer, using the double emulsion method. The reason that a PEGylated PLGA multicomponent system was designed is that the addition of PEG on the surface of the PLGA nanoparticles can give the targeted multicomponent drug delivery system a stealth barrier, which can delay opsonization and recognition by the reticuloendothelial system [67]. In vitro release investigations showed that the OXA from the multicomponent drug delivery systems, PLGA_IO-OA_OXA and PEGylated PLGA_IO-OA_OXA, were faster for the PEGylated analog. The concentration of the released OXA from the PEGylated PLGA_IO-OA_OXA increased very rapidly, reaching ~100% release after only 2 h, while the PLGA_IO-OA_OXA displayed a slower and sustained drug release. The super-fast and non-controlled OXA release in the PEGylated PLGA_IO-OA_OXA is probably connected with the hydrophilic nature of OXA, the PEGylation of the nanoparticles, and the presence of IO-OA. A similar observation of a quicker release, using a hydrophilic carrier (PEG-PLGA), was observed in the work of Babos et al., in which a water-soluble drug (doxorubicin HCl) was encapsulated into both PLGA-based and PEG–PLGA nanoparticles [68]. Therefore, for a targeted system with controlled release, the PLGA_IO-OA_OXA was chosen for the conjugation with the anti-CD133 antibody, conjugated to Alexa Fluor 488.

Based on the expression profile of CD133 in CaCo-2 cells [52], we utilized the interaction of anti-CD133 Ab-coated nanoparticles and CD133 cell surface antigens as a very promising approach to realize the highly specific and effective delivery of OXA to CD133-overexpressing cells from colorectal carcinoma, which is difficult to treat efficiently. The reason why the CD133 (prominin-1) antigen, a surface transmembrane glycoprotein, was chosen for this study is that it is one of the most frequently used cell surface antigens for detection and isolation of cancer stem cells, from various solid tumors [69], including the brain, colon, pancreas, prostate, lung, and liver. Among others, its expression has been documented also for CaCo-2 cells [52]. The prepared PLGA_OXA_Ab and PLGA_IO-OA_OXA_Ab in this study demonstrated the proper interaction of the nanoparticles with the CD133 cell surface antigen, which can confirm the delivery of the multicomponent system and can be used as a drug delivery of OXA, as the metabolic assay showed the elimination of the cancer cells.

## 4. Materials and Methods

### 4.1. Materials

Iron (III) chloride hexahydrate (≥99%), iron (II) chloride tetrahydrate (98%), oleic acid (≥90%) (OA), potassium dihydrogen phosphate (≥98%), N-hydroxysuccinimide (NHS) (98%), N-(3-dimethyl aminoproyl)-*N’*-ethylcarbodiimide hydrochloride (EDC), *N,N*′-dicyclohexylcarbodiimide (DCC) (≥99.0%), poly(ethylene glycol diamine) (NH_2_-PEG-NH_2_, Mn = 3000), (≥98.0%), polyvinyl alcohol (PVA, Mowiol 4–88), deuterated chloroform (≥99%) and oxaliplatin (OXA) (≥99%) were purchased from Sigma-Aldrich (St. Louis, MO, USA). Dialysis bags (Spectra/Por, MWCO: 3.5 and 12–14 kDa) and a monoclonal anti-CD133-TMP4 Ab conjugated to Alexa Fluor 488 were purchased from Thermo Fisher Scientific (Waltham, MA, USA). Commercial PLGA Mn of 10 kDa (Purasorb PDLG 7502A, 70/30 LA/GA) was kindly donated by Corbion (The Netherlands). Sodium hydroxide, chloroform, ethyl ether, tetrahydrofuran (THF), dichloromethane (DCM), ethanol, and ammonia p.a. were purchased from Penta (The Czech Republic). Media used for cell culture Eagle’s Minimum Essential Medium (EMEM) and Dulbecco’s Modified Eagles’ medium (DMEM) were bought from Thermo Fisher Scientific (Waltham, MA, USA). Microscopy dishes with glass bottom (ø35 mm, 1.5#) were supplied by MatTek Life Sciences (Boston, MA, USA). Filters with a 0.45-µm pore size were purchased from JetBiofil (China). For all experiments, Millipore water was used.

### 4.2. Synthesis of Iron Oxide Nanoparticles

Synthesis of IO NPs coated with oleic acid (IO-OA NPs) was achieved by co-precipitation using the protocol described in our previous work [33]. Briefly, iron (II) chloride tetrahydrate and iron (III) chloride hexahydrate at a molar ratio of 1:2 were incorporated in degassed Millipore water. To initiate the co-precipitation reaction, ammonium hydroxide was added to the mixture. Oleic acid was added to the reaction system, which was then heated to 80 °C and left for magnetic stirring for 1 h. Thereafter, the IO-OA suspension was washed several times before drying in the oven and redispersed in chloroform. The final concentration of IO-OA in the suspension was set to 10 mg·mL^−1^.

### 4.3. Synthesis of PLGA-PEG-NH_2_ Copolymer

Synthesis of the amine-terminated diblock copolymer was prepared based on the protocol described in our previous work [33]. In short, PLGA was first activated using NHS and DCC using carbodiimide chemistry. The reaction was left for magnetic stirring for 20 h at room temperature (25 °C). Insoluble dicyclohexyl urea was filtered, followed by precipitation of the polymer using ice-cold diethyl ether. The activated PLGA was dried under vacuum at room temperature (25 °C), followed by the conjugation with the excess of NH_2_-PEG-NH_2_ to avoid the formation of triblock copolymer. The amine-terminated diblock copolymer was precipitated and washed with ice-cold methanol. The final PLGA-PEG-NH_2_ polymer was dried under vacuum at room temperature (25 °C).

### 4.4. Preparation of the Multicomponent Drug Delivery Systems

The multicomponent drug delivery systems were prepared by the double emulsion method described by Moreno et al. [42], with slight modifications, namely, PLGA_IO-OA_OXA and PEGylated PLGA_IO-OA_OXA. Fifty milligrams of PLGA or PLGA-PEG-NH_2_ were dissolved in 1.5 mL of chloroform, followed by the addition of 5 mg of the IO-OA suspension (10 mg·mL^−1^). Oxaliplatin (5 mg) was dissolved in 1 mL of Millipore water and it was added to the polymer/IO-OA organic solution and was emulsified for 5 min at 40 W on an ice-bath using a sonication probe (Sonopuls HD 450, Bandelin) with ON/OFF states of 10 s each. The resulting emulsion was transferred into an aqueous solution of PVA 3% (*w*/*w*) (6 mL) and it was emulsified by sonication at 40 W for 5 min with ON/OFF states of 10 s each. The final emulsion was diluted to a final concentration of 0.6 % (*w*/*v*) PVA. Finally, the suspension was left for magnetic stirring for 24 h to remove the organic solvent. It was then purified by dialysis for 48 h (molecular weight cut off 12–14 kDa) against 600 mL of Millipore water (with frequent changes of Millipore water). The final nanoparticle suspension was filtered through a 5 µm filter to remove the dust. The final concentration of all nanoparticles was set to 1.8 mg·mL^−1^. The ratio of oxaliplatin to polymer and IO-OA to polymer was set to 1:10 (*w*/*w*) [33]. It is worth noting that the ratio of IO-OA polymer was set to 1:10 (*w*/*w*) based on our previous work, in which the effect of the ratio of IO-OA to polymer on the encapsulation efficiency, mean diameter, and the magnetic properties of the loaded nanoparticles was investigated and optimized [33]. For the sake of fundamental understanding of the behavior of multicomponent loaded delivery systems, polymeric nanoparticles loaded with a single therapeutic agent were also prepared following the same procedure, namely, PLGA_IO-OA, PLGA_OXA, PEGylated PLGA_IO-OA, and PEGylated PLGA_OXA.

### 4.5. Antibody Conjugation

Conjugation of the monoclonal anti-CD133-TMP4 Ab conjugated to Alexa Fluor 488 on the surface of PLGA_IO-OA_OXA was performed using carbodiimide chemistry. The amount of 10 mg of PLGA_IO-OA_OXA aqueous suspension (1.8 mg·mL^−1^) was centrifuged (21,000 g, 10 min, 4 °C) to replace the Milli-Q water media with 10 mL of 10mM PBS of pH 5.8. The surface of PLGA_IO-OA_OXA NPs was activated by adding 100 µL of NHS (0.7 M) and 100 µL of EDC (0.1 M) to the suspension. The reaction medium was left for moderate stirring (100 rpm) for 1 h at room temperature (25 °C). The activated nanoparticles were then centrifuged (21,000 g, 10 min, 4 °C) and washed three times with 10 mM PBS (pH 7.4) followed by their suspension in 10 mL of PBS before the antibody conjugation. Next, 400 µL (0.25 µg mL^−1^) of the anti-CD133-TMP4 Ab conjugated to Alexa Fluor 488 was then added to 1 mL (1.8 mg·mL^−1^) of the activated nanoparticles, placed in a dark Eppendorf tube and the reaction medium was left for moderate stirring for 4 h. To quench the reaction, 8 µL of glycine (0.2 mM) was added to the conjugated nanoparticles with moderate stirring for 5 min. Finally, the conjugated nanoparticles were purified using a PD10 column (Cytiva [originally GE Healthcare], Marlborough, MA, USA) and suspended in storage buffer PBS (pH 7.4, 10 mM) with 0.02% NaN_3_. The conjugated nanoparticles were labeled as PLGA_IO-OA_OXA_Ab.

### 4.6. Drug Loading and Dissolution Test

The drug loading content which represents the weight fraction of oxaliplatin captured in the nanoparticles after the processing was determined by means of thermogravimetric analysis (TGA, Stanton-Redcroft TG 750). TGA were performed under nitrogen atmosphere (20 mL·min^−1^) from room temperature (25 °C) to 800 °C at a rate of 10 °C·min^−1^

The encapsulation efficiency (EE (%)) was calculated as follows:

(1) EE (%) = [(amount of encapsulated oxaliplatin)/ (amount of oxaliplatin added)]* 100,

To carry out the dissolution tests, 10 mg of the freeze-dried nanoparticles were suspended in 5 mL of PBS (0.1 M, pH 7.4) in a dialysis bag (molecular weight cutoff 3.5 kDa). The suspensions were then incubated in 35 mL of PBS at 37 °C under magnetic stirring at 100 rpm. At specific intervals, 1.5 mL of sample was taken from the PBS bath and refilled with the same volume of fresh PBS. The concentration of released oxaliplatin as a function of time was monitored using UV-visible spectrophotometer at a wavelength of 200 nm based on a calibration curve. All experiments were reproduced in triplicates.

### 4.7. In Vitro Studies in Human Cells

The suspensions of PLGA_OXA_Ab and PLGA_IO-OA_OXA_Ab were sterilized by filtration using a 0.45-µm filter and UV-light irradiation for 20 min. The following cell lines were used for the in vitro experiments: CaCo-2 (human cells derived from colon carcinoma), and MRC-5 cells (human primary noncancerous cells); all cells were from the American Tissue Culture Collection. The cells were cultured in the following cultivation media: CaCo-2 cells in EMEM supplemented with 20% fetal bovine serum (FBS), and MRC-5 cells in EMEM with 10 % FBS. The cells were maintained at the exponential growth phase and, for the experiments, they were seeded on 35-mm microscopic glass-bottom dishes in a sterile hood with laminar flow. Next, 100,000 cells were seeded per one dish in 2 mL of cultivation media and incubated at standard experimental conditions (37 °C, 5 % CO_2_, and 95% humidity) in a sterile incubator. At 16 h after seeding, the cell culture medium was removed and the cells were washed with PBS, which was then exchanged with 2 mL of fresh media with the addition of the tested nanoparticles (PLGA_IO-OA_OXA_Ab) at the following amounts: 2 µL (3.6 µg), 20 µL (36 µg), and 100 µL (180 µg). The stock concentration of the nanoparticles was 1.8 mg·mL^−1^. Then, the cells were incubated in the incubator for 30 min and subjected to fluorescence microscopy. In some cases, the cells were stained or co-stained with anti-CD133 conjugated to Atto 565 (2 µL of the antibody per 2 mL of the cell sample for 30 min.). Untreated cells incubated only in media free of nanoparticles served as a negative control.

#### 4.7.1. Sample Fixation

In addition to living cells, fixed samples were also prepared. The procedure corresponds to that described in Section 4.7. Then, after the samples’ incubation with the nanoparticles, the medium was removed, the samples were twice washed with PBS, fixed with a 4% solution of methanol-free formaldehyde (Thermo Fisher Scientific, Waltham, MA, USA) in PBS for 20 min at 22 °C in the dark. Then, the samples were washed twice by PBS and kept in dark before fluorescence microscopy.

#### 4.7.2. Fluorescence Microscopy

PLGA_IO-OA_OXA_Ab binding to cells of the examined cell lines was performed by wide-field fluorescence microscopy of both live and fixed samples using an Olympus IX-81 microscope (Olympus, Tokyo, Japan). The images were acquired using EM-CCD camera C9100-02 (Hamamatsu, Japan) by xCellence software. The 60x oil immersion objective with an NA of 1.4 was used. For sample excitation, a high-stability 150 W xenon burner was applied in combination with an excitation/emission FITC and TRITC filter to visualize Alexa Fluor 488 and Atto 565, respectively. The images were background-corrected and deconvolved by two-dimensional deconvolution with the no-neighbor algorithm (Olympus, Tokyo, Japan).

#### 4.7.3. Metabolic Activity Assay

The impact of the prepared nanoparticles with OXA on cell viability was examined in vitro using WST-1 assay (Sigma-Aldrich, St. Louis, MO, USA). For each cell line, 5000 to 10,000 cells (depending on the specific cell line) per well of 96 wells was seeded into single wells in 100 µL of complete cell cultivation media. Then, the cells were cultivated for 16 h at standard cultivation conditions, after which they were treated with 100 µL of fresh cell cultivation media containing the evaluated nanoparticles. Cell viability was then determined 72 h after cell treatment by the following procedure: the culture medium was discarded and changed for 100 µL of fresh phenol red-free DMEM with the addition of 5 µL of WST-1 reagent. After that, the cells were incubated for an additional 2 h in the incubator. Then, the absorbance (of formazan) was measured spectrophotometrically at 450 nm (ref. wavelength of 630 nm) using a UV-Vis spectrometer (Bio-Rad Laboratories, Hercules, CA, USA). All samples were measured in quadruplicates. Untreated cells and cells treated only with blank (pure nanoparticles) served as controls.

### 4.8. Characterization

#### Dynamic Light Scattering

For dynamic light scattering measurement, the particle size distribution was measured using a Zetasizer Nano ZS (Malvern Instruments, Malvern, Great Britain). The amount of 1 mL of each suspension was placed in a cuvette without further dilution. All measurements were performed in triplicate in an autocorrelation mode with a single illuminating beam and detector with a scattering angle of 90° at room temperature (25 °C).

#### Zetasizer Potential

The zeta potential of the prepared nanoparticles was measured using Zetasizer Nano ZS (Malvern Instruments, Malvern, Great Britain) using the Smoluchowski model. The measurements were carried out on 800 µL of 10× times diluted aqueous mother suspension. Measurement of the zeta potential of IO-OA was performed by dispersing 28 µL of IO-OA suspension in 1 mL of Milli-Q water and the removal of chloroform by evaporation before the analysis. It is worth noting that the colloidal stability of IO-OA in an aqueous solution was retained during the measurement. All measurements were performed in triplicates at room temperature (25 °C).

#### Transmission Electron Microscopy

The morphology of the prepared nanoparticles was characterized by Transmission Electron Microscopy (TEM). The measurements were carried out using 100 kV TEM, model JEM-1010 (JEOL, Ltd.) equipped with CCD camera MegaView III (Olympus Soft Imaging Systems, Germany). In short, 10 µL of suspension was deposited on a carbon-coated electron microscopic grid. Sample contrasting was performed using 1% (*w/w*) uranyl acetate. The grid was dried and then was introduced into the electron microscope column for analysis.

#### Attenuated Total Reflection Fourier-Transformed Infrared Spectroscopy

Thermo Scientific Nicolet iZ10 Fourier transform infrared spectrometer in attenuated total reflection mode (ATR-FTIR) with a diamond crystal was utilized to identify the molecular structure of the PLGA based copolymers and the drug delivery systems. The FTIR spectra are an average of 64 scans.

#### Thermogravimetric Analysis

Thermogravimetric analyses (TGA) were carried out using Stanton Redcroft TG 750 under nitrogen atmosphere (20 mL·min^−1^) from room temperature (25 °C) to 800 °C at a rate of 10 °C·min^−1^ to determine the drug loading content of OXA captured in the nanoparticles during the processing.

#### Vibrating Sample Magnetometer

The magnetic properties of the multicomponent drug delivery systems were assessed by a vibrating sample magnetometer (VSM 7407, Lake Shore, MD, USA) at room temperature (25 °C) in the magnetic field from −10 kOe to +10 kOe.

### 4.9. Statistical Analysis

All measurements were performed in triplicates and were expressed as means with standard deviation. Differences between nanoparticles were tested using a one-way ANOVA with a level of significance of *p* ≤ 0.05.

## 5. Conclusions

In this study, Ab conjugated PLGA nanocarriers for the targeted delivery of OXA and superparamagnetic nanoparticles to colorectal cancer cells were developed with the aim of suppressing the IPN caused by cumulative doses of OXA, and to enhance the treatment efficacy. Co-encapsulation of OXA and IO-OA was achieved in two polymeric carriers, namely, PLGA and PLGA-PEG, using the double emulsion method. PLGA_IO-OA_OXA and PEGylated PLGA_IO-OA_OXA displayed a comparable mean diameter of 207 ± 70 nm and 185 ± 119 nm, respectively. The encapsulation of OXA and/or IO-OA and the PEGylation process did not statistically affect the hydrodynamic size of the nanoparticles. The concentration of the released OXA from the PEGylated PLGA_IO-OA_OXA increased very rapidly, reaching ~100% release after only 2 h, while the PLGA_IO-OA_OXA displayed a slower and sustained drug release. For a controlled release of OXA, non-PEGylated PLGA nanoparticles are thus more suitable. Preservation of the superparamagnetic behavior of the IO-OA without magnetic hysteresis all along the dissolution process was also pointed out, thereby making the multicomponent drug delivery systems suitable for treatment with magnetically mediated hyperthermia, even after the drug release is complete.

To specifically target cancer cells overexpressing CD133, the non-PEGylated nanoparticles were conjugated to anti-CD133 Alexa Fluor 488 Ab. The Ab-coated nanoparticles were efficiently bound to CD133 expressing cells, as documented in the CaCo-2 cells. The OXA-containing nanoparticles were able to eliminate the cancer cells, which was shown using a metabolic activity assay. Therefore, the prepared nanoparticles are suitable not only for specific cancer cell targeting and selective drug delivery, but also for the imaging of such cells and their eradication. The concept is expandable and could also be used for the delivery of other anticancer drugs.

## Figures and Tables

**Table 1:** Average diameter and zeta potential of the prepared nanoparticles.

Sample Name	Mean Diameter (nm)	PDI *	Zeta Potential (mV)
IO-OA	20 ± 4	0.13 ± 0.07	−36 ± 4
PLGA	245 ± 86	0.05 ± 0.03	−9 ± 4
PLGA_IO-OA	220 ± 89	0.08 ± 0.02	−8 ± 6
PLGA_OXA	190 ± 59	0.06 ± 0.003	−5 ± 6
PLGA_OXA_Ab	285 ± 74	0.191 ± 0.026	−3 ± 4
PLGA_IO-OA_OXA	207 ± 70	0.08 ± 0.006	−8 ± 7
PLGA_IO-OA_OXA_Ab	223 ± 70	0.136 ± 0.026	−4 ± 5
PEGylated PLGA	146 ± 50	0.1 ± 0.01	0 ± 4
PEGylated PLGA_OXA	130 ± 51	0.2 ± 0.009	5 ± 4
PEGylated PLGA_IO-OA	217 ± 160	0.2 ± 0.014	2 ± 4
PEGylated PLGA_IO-OA_OXA	185 ± 119	0.2 ± 0.004	4 ± 3

* Polydispersity index (PDI) with standard deviation (from 3 replicates).

**Table 2:** Evaluation of in vitro metabolic activity of human cells derived from colorectal carcinoma (CaCo-2) and human primary fibroblasts (MRC-5) treated with PLGA_OXA and PLGA_IO_OXA for 72 h determined by WST-1 assay. IC_50_—concentration of the nanoparticles required to achieve 50 % reduction in cell viability; SD—standard deviation (from four replicates).

	IC_50_ ± SD [µg·mL^−1^] of the Nanoparticles	
Cell line	PLGA_OXA	PLGA_IO_OXA
CaCo-2	255 ± 11	560 ± 62
MRC-5	525 ± 12	325 ± 31

**Table 3:** Evaluation of in vitro metabolic activity of human cells derived from colorectal carcinoma (CaCo-2) and human primary fibroblasts (MRC-5) treated with PLGA_OXA and PLGA_IO_OXA for 72 h determined by WST-1 assay. IC_50_—concentration of the oxaliplatin (free—OXA, and in the nanoparticles) required to achieve 50 % reduction in cell viability; SD—standard deviation (from four replicates).

	IC_50_ ± SD [µg·mL^−1^] of the Oxaliplatin		
Cell line	PLGA_OXA	PLGA_IO_OXA	OXA
CaCo-2	5.66 ± 0.24	12.44 ± 1.38	8.35 ± 0.48
MRC-5	11.66 ± 0.27	7.22 ± 0.69	14.30 ± 1.26

## Data Availability

The data presented in this study are available on request from the corresponding author.

## Supplementary Materials

The following supporting information can be downloaded at: https://www.mdpi.com/article/10.3390/ijms23031200/s1.

## Abbreviations

^1^H-NMR	Proton Nuclear Magnetic Resonance
5-FU	5-Fluorouracil
Ab	Antibody
anti-CD133-TMP4	Monoclonal antibody CD133-TMP4 conjugated to Alexa Fluor 488
CaCo-2	Human cells derived from colorectal carcinoma
CSC	Cancer stem cells
DCC	*N,N*′-dicyclohexylcarbodiimide
DCM	Dichloromethane
DLS	Dymanic Light Scattering
DMEM	Dulbecco’s Modified Eagles’ medium
DNA	Deoxyribonucleic acid
EDC	*N*-(3-dimethyl aminoproyl)-*N*′-ethylcarbodiimide hydrochloride
EMEM	Eagle’s Minimum Essential Medium
FTIR	Fourier-transform Infrared spectroscopy
IO	Iron Oxide nanoparticles
IO-OA	Oleic acid-coated iron oxide nanoparticles
IPN	Induced peripheral neurotoxicity
MRC-5	Noncancerous human primary fibroblasts
MRI	Magnetic Resonance Imaging
Ms	Saturation magnetization
NH_2_-PEG-NH_2_	Poly(ethylene glycol diamine)
NHS	*N*-hydroxysuccinimide
OA	Oleic acid
OXA	Oxaliplatin
PBS	Phosphate Buffer Saline
PDI	Polydispersity index
PEGylated PLGA	PEGylated poly(lactide-co-glycolide) nanoparticles
PEGylated PLGA_IO-OA	Oleic acid-coated iron oxide-loaded PEGylated poly(lactide-co-glycolide) nanoparticles
PEGylated PLGA_IO-OA_OXA	Oleic acid-coated iron oxide and oxaliplatin-loaded PEGylated poly(lactide-co-glycolide) multicomponent delivery system
PEGylated PLGA_OXA	Oxaliplatin-loaded PEGylated poly(lactide-co-glycolide) nanoparticles
PLGA	Poly(lactide-co-glycolide)
PLGA_IO-OA	Oleic acid-coated iron oxide-loaded poly(lactide-co-glycolide) nanoparticles
PLGA_IO-OA_OXA	Oleic acid-coated iron oxide and oxaliplatin-loaded poly(lactide-co-glycolide) multicomponent delivery system
PLGA_IO-OA_OXA_Ab	anti-CD133 antibody conjugated oleic acid-coated iron oxide and oxaliplatin-loaded poly(lactide-co-glycolide) multicomponent delivery system
PLGA_OXA	Oxaliplatin-loaded poly(lactide-co-glycolide) nanoparticles
PLGA_OXA_Ab	anti-CD133 antibody conjugated oxaliplatin-loaded poly(lactide-co-glycolide) nanoparticles
PLGA-PEG-NH_2_	Poly(lactide-co-glycolide)-poly(ethylene glycol) bearing amino end group
PVA	Polyvinyl alcohol
RNA	Ribonucleic acid
SD	Standard Deviation
SEC	Size Exlcusion Chromatography
TEM	Transmission Electron Microscopy
TGA	Thermogravimetric Analysis
THF	Tetrahydrofuran
VSM	Vibrating Sample Magnetometer
XRD	X-ray Diffraction
