# Supplementary material for: Antibody Conjugated PLGA Nanocarriers and Superparmagnetic Nanoparticles for Targeted Delivery of Oxaliplatin to Cells from Colorectal Carcinoma
Source: Int J Mol Sci. 2022 Jan 21;23(3):1200. doi: 10.3390/ijms23031200 (PMC8835878; doi:10.3390/ijms23031200)
Supplement: Supplementary file 1 [file ijms-23-01200-s001.zip › ijms-1503617-supplementary.pdf]

International Journal of *Molecular Sciences*

MDPI

Supporting Information

Antibody Conjugated PLGA Nanocarriers and Superparmagnetic Nanoparticles for Targeted Delivery of Oxaliplatin to Cells from Colorectal Carcinoma

Alma Lucia Villela Zumaya [1], Silvie Rimpelová [2], Markéta Štějdířová [1], Pavel Ulbrich [2], Jarmila Vilčáková [3] and Fatima Hassouna [1,*]

[1] Faculty of Chemical Engineering, University of Chemistry and Technology Prague, 166 28 Prague 6, Czech Republic; zumayaa@vscht.cz (A.L.V.Z.); stejdirm@vscht.cz (M.Š.)
[2] Faculty of Food and Biochemical Technology, University of Chemistry and Technology Prague, 166 28 Prague 6, Czech Republic; silvie.rimpelova@vscht.cz (S.R.); ulbrichp@vscht.cz (P.U.)
[3] Faculty of Technology, Tomas Bata University, 760 01 Zlín, Czech Republic; vilcakova@utb.cz
* Correspondence: fatima.hassouna@vscht.cz (F.H.); Tel.: +420-220-444-099

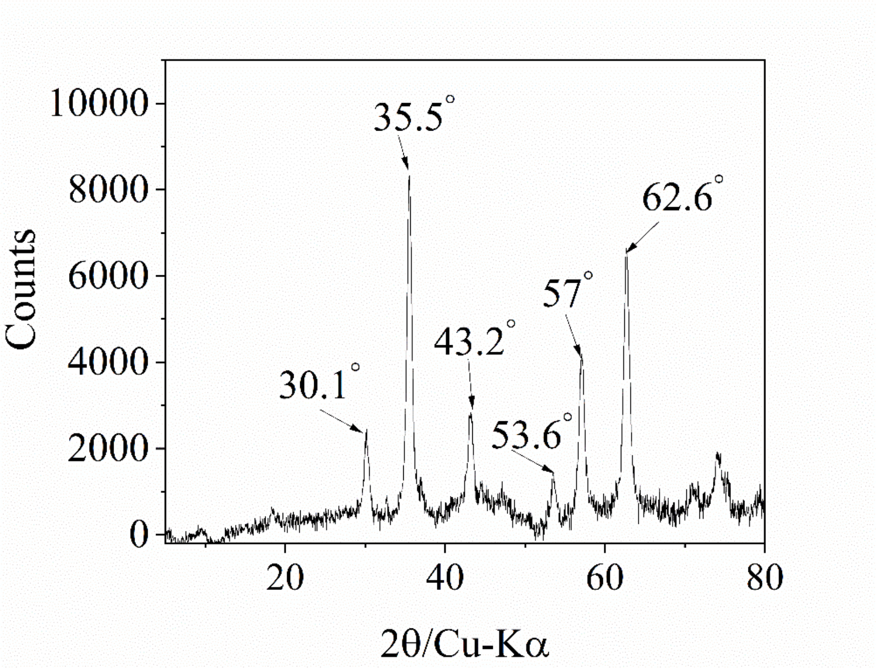

Figure S1. X-ray diffraction pattern of superparamagnetic oleic-acid coated iron oxide nanoparticles (IO-OA).

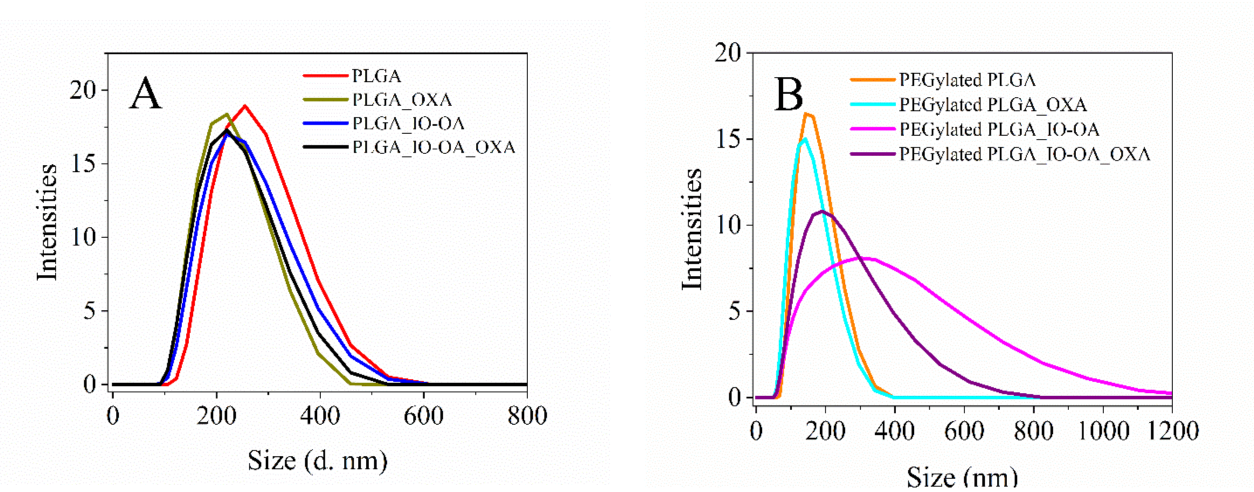

Figure S2. Particle size distribution of A) poly(lactide-co-glycolide) nanoparticles (PLGA), oxaliplatin loaded PLGA nanoparticles (PLGA_OXA), oleic acid-coated iron oxide loaded PLGA nanoparticles (PLGA_IO-OA) and PLGA based multicomponent delivery systems (PLGA_IO-OA_OXA), B) poly(lactide-co-glycolide)-poly(ethylene glycol) nanoparticles (PEGylated PLGA), oxaliplatin loaded PEGylated nanoparticles (PEGylated PLGA_OXA), oleic-acid coated iron oxide loaded PEGylated PLGA nanoparticles (PEGylated PLGA_IO-OA) and PEGylated PLGA based multicomponent delivery system (PEGylated PLGA_IO-OA_OXA).

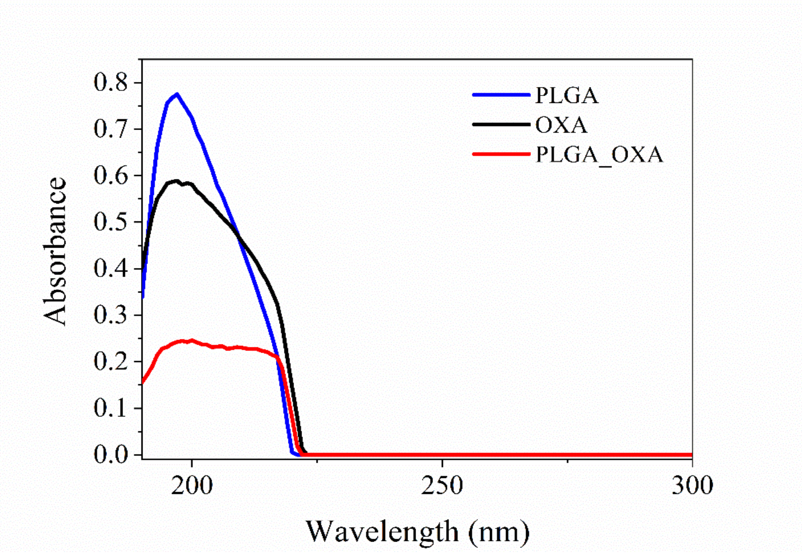

Figure S3. UV-Vis spectra of poly(lactide-co-glycolide) nanoparticles (PLGA), oxaliplatin (OXA) and oxaliplatin loaded PLGA nanoparticles (PLGA_OXA) in PBS pH 7.4.

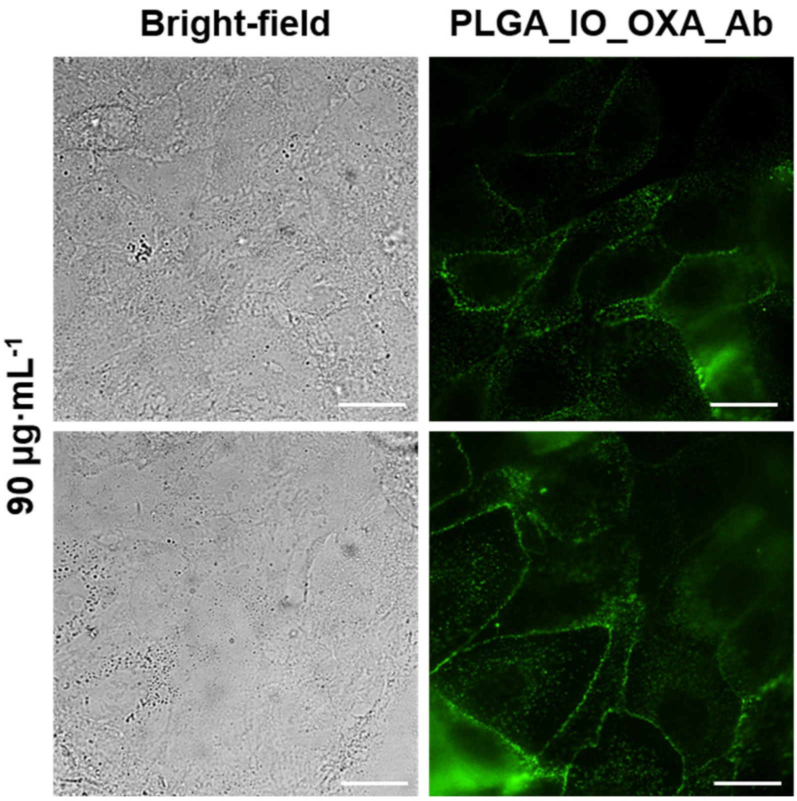

Figure S4. Fluorescence microscopy images of human cells derived from colorectal carcinoma treated with iron oxide and oxaliplatin-containing PLGA nanoparticles coated with anti-CD133 antibody conjugated to Alexa Fluor 488 (PLGA_OXA_Ab). The Caco-2 cells were treated with 90 µg·mL-1 concentration of the PLGA_IO_OXA_Ab for 30 min. Left – bright-field images of the cells, right – fluorescence emission of cells treated with PLGA_IO_OXA_Ab. The scale bars correspond to 20 µm.

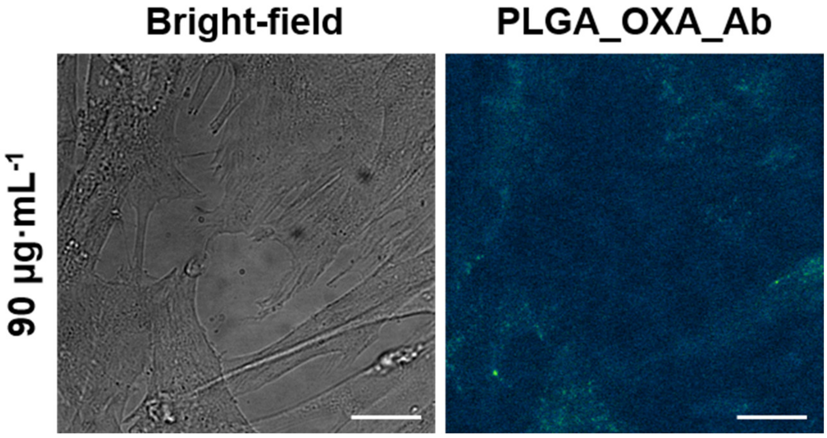

Figure S5. Fluorescence microscopy images of human primary fibroblasts (MRC-5) treated with oxaliplatin-containing PLGA nanoparticles coated with anti-CD133 antibody conjugated to Alexa Fluor 488 (PLGA_OXA_Ab). The MRC-5 cells were treated with 90 µg·mL-1 concentration of the PLGA_OXA_Ab for 30 min. Left – a bright-field image of the cells, right – fluorescence emission of cells treated with PLGA_OXA_Ab (false coloured based on the fluorescence emission intensity). The scale bars correspond to 20 µm.

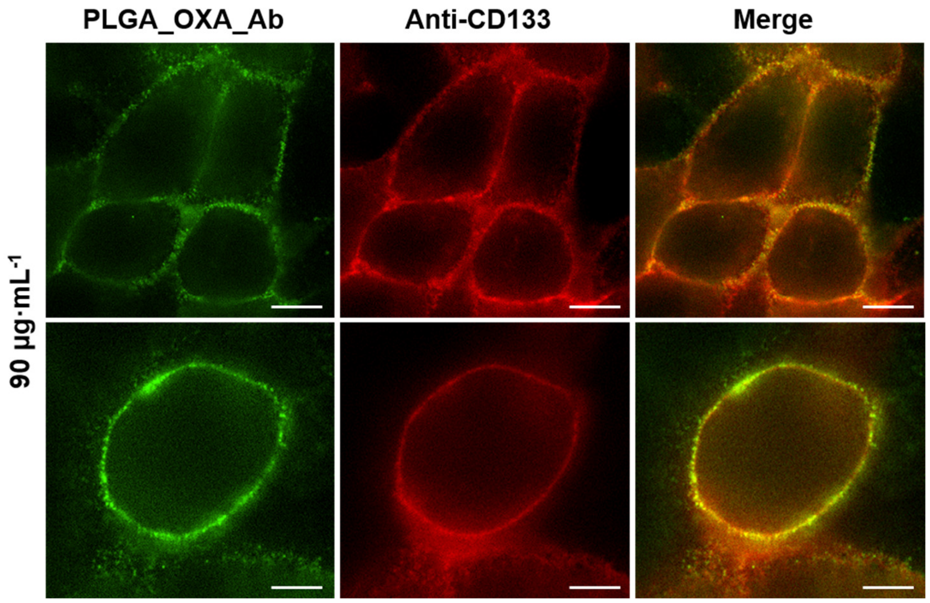

Figure S6. Fluorescence microscopy images of human cells derived from colorectal carcinoma (CaCo-2) treated with oxaliplatin-containing PLGA nanoparticles coated with anti-CD133 antibody conjugated to Alexa Fluor 488 (PLGA_OXA_Ab) and co-stained with anti-CD133 Atto 565. The CaCo-2 cells were treated with 90 μg·mL-1 concentration of the PLGA_OXA_Ab for 30 min. From left: bright-field images of the cells; fluorescence emission of cells treated with PLGA_OXA_Ab; cells stained with anti-CD133-Atto 565; and merge of the fluorescence images. The scale bars correspond to 20 μm.

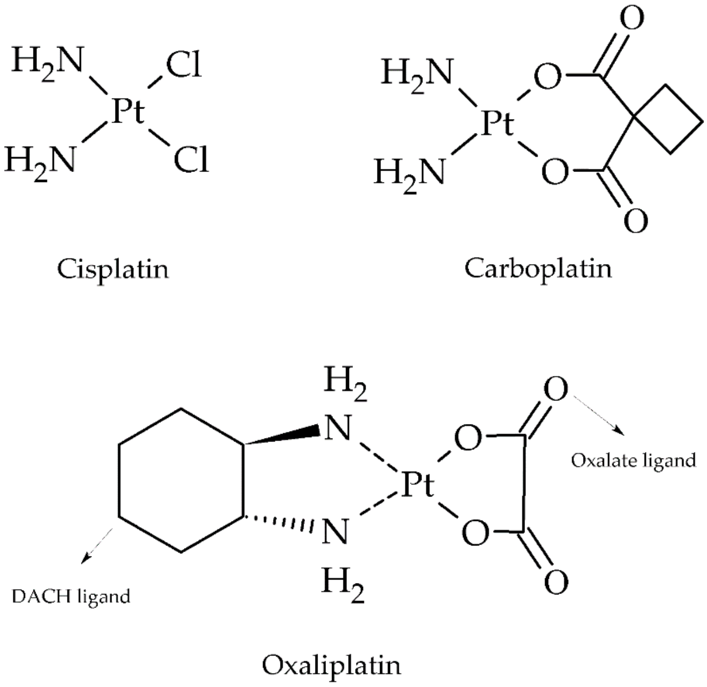

Figure S7. Chemical structure of platinum compounds used for treatment of cancer.

Table S1. Average mean diameter (nm) based on TEM using ImageJ software for the prepared nanoparticles.

Sample Name	Mean Diameter (nm) by TEM
PLGA	110 ± 60
PLGA_IO-OA	118 ± 41
PLGA_OXA	108 ±28
PLGA_OXA_Ab	175 ± 40
PLGA_IO-OA_OXA	119 ± 22
PLGA_IO-OA_OXA_Ab	166 ± 25
PEGylated PLGA	100 ± 23
PEGylated PLGA_OXA	92 ± 14
PEGylated PLGA_IO-OA	100 ± 18
PEGylated PLGA_IO-OA_OXA	97 ± 51

Table S2. Drug release values as cumulative concentration (μg·ml^{-1}) over time for the oxaliplatin-loaded PLGA nanoparticles, PLGA-based multicomponent delivery system and their PEGylated analogs.

Time (h)	Cumulative Concentration (μg ·ml^{-1})			
	PLGA_OXA ± SD	PLGA_IO-OA_OXA ± SD	PEGylated PLGA_ OXA ± SD	PEGylated PLGA_IO-OA_OXA ± SD
0.083	0.28 ± 0.28	0.78 ± 0.17	0.18 ± 0.18	3.68 ± 0.51
0.25	0.28 ± 0.29	3.36 ± 1.17	2.21 ± 0.99	8.65 ± 3.14
0.33	0.28 ± 0.28	6.93 ± 1.61	3.85 ± 0.76	11.44 ± 3.13
0.5	0.28 ± 0.28	9.57 ± 3.16	4.27 ± 1.18	16.28 ± 4.13
0.66	0.89 ± 0.89	11.71 ± 2.97	5.23 ± 2.14	20.31 ± 2.70
0.833	0.89 ± 0.88	-	8.20 ± 3.54	23.94 ± 2.96
1	1.21 ± 0.56	14.58 ± 3.10	11.72 ± 3.52	35.16 ± 4.98
1.5	1.21 ± 0.53	-	14.21 ± 4.99	40.54 ± 7.05
2	2.15 ± 1.49	18.95 ± 2.37	17.59 ± 5.17	43.23 ± 7.89
3	2.40 ± 1.74	22.23 ± 3.1	22.78 ± 7.07	
4	-	25.75 ± 1.64	32.34 ± 7.28	
24	6.68 ± 0.43	28.33 ± 0.61	-	
48	-	32.03 ± 1.92	-	
72	-	34.72 ± 2.26	-	
96	8.91 ± 0.17	37.46 ± 3.01	41.84 ± 7.40	
120	16.96 ± 0.73	-		
144	22.08 ± 1.05	-		
168	26.92 ± 1.25	40.39 ± 3.59		
192	34.64 ± 4.92			
264	41.78 ± 3.85			
